# 3-({[(1-Phenyl­eth­yl)sulfan­yl]methane­thio­yl}sulfan­yl)propanoic acid

**DOI:** 10.1107/S1600536811046873

**Published:** 2011-11-19

**Authors:** M. Kannan, V. Ramkumar, R. Dhamodharan

**Affiliations:** a Department of Chemistry, IIT Madras, Chennai, Tamil Nadu, India

## Abstract

In the title compound, C_12_H_14_O_2_S_3_, a chain transfer agent (CTA) used in polymerization, the dihedral angle between the aromatic ring and the CS_3_ grouping is 84.20 (10)°. In the crystal, carb­oxy­lic acid inversion dimers linked by pairs of O—H⋯O hydrogen bonds generate *R*_2_^2^(8) loops.

## Related literature

For background to chain transfer agents, see: Chong *et al.* (1999 ▶); Coady *et al.* (2008 ▶). For a related structure, see: Kannan *et al.* (2010 ▶).
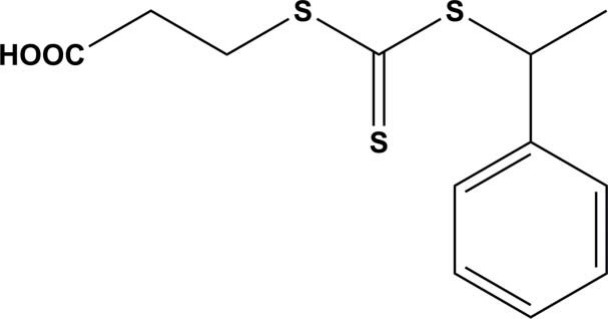


## Experimental

### 

#### Crystal data

C_12_H_14_O_2_S_3_
*M* *_r_* = 286.41
Monoclinic, 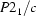

*a* = 13.6280 (8) Å
*b* = 10.2908 (5) Å
*c* = 10.7299 (5) Å
β = 113.039 (2)°
*V* = 1384.77 (12) Å^3^
*Z* = 4
Mo *K*α radiation
μ = 0.52 mm^−1^
*T* = 298 K
0.42 × 0.28 × 0.22 mm

#### Data collection

Bruker APEXII CCD area-detector diffractometer
Absorption correction: multi-scan (*SADABS*; Bruker, 2004 ▶) *T*_min_ = 0.811, *T*_max_ = 0.894
9109 measured reflections
3020 independent reflections
2224 reflections with *I* > 2σ(*I*)
*R*_int_ = 0.017

#### Refinement

*R*[*F* ^2^ > 2σ(*F* ^2^)] = 0.036
*wR*(*F* ^2^) = 0.095
*S* = 1.03
3020 reflections
159 parameters
H atoms treated by a mixture of independent and constrained refinement
Δρ_max_ = 0.32 e Å^−3^
Δρ_min_ = −0.27 e Å^−3^

### 

Data collection: *APEX2* (Bruker, 2004 ▶); cell refinement: *SAINT-Plus* (Bruker, 2004 ▶); data reduction: *SAINT-Plus*; program(s) used to solve structure: *SHELXS97* (Sheldrick, 2008 ▶); program(s) used to refine structure: *SHELXL97* (Sheldrick, 2008 ▶); molecular graphics: *ORTEP-3* (Farrugia, 1997 ▶); software used to prepare material for publication: *SHELXL97*.

## Supplementary Material

Crystal structure: contains datablock(s) global, I. DOI: 10.1107/S1600536811046873/hb6480sup1.cif

Structure factors: contains datablock(s) I. DOI: 10.1107/S1600536811046873/hb6480Isup2.hkl

Supplementary material file. DOI: 10.1107/S1600536811046873/hb6480Isup3.cml

Additional supplementary materials:  crystallographic information; 3D view; checkCIF report

## Figures and Tables

**Table 1:** Hydrogen-bond geometry (Å, °)

*D*—H⋯*A*	*D*—H	H⋯*A*	*D*⋯*A*	*D*—H⋯*A*
O2—H1*O*⋯O1^i^	0.81 (3)	1.85 (3)	2.651 (2)	177 (3)

Symmetry code: (i) 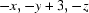.

**Fig. 1.**
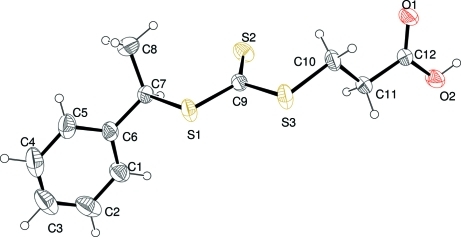
The molecular structure of the title molecule with atoms represented as 30% probability ellipsoids.

## supplementary crystallographic information

### Comment

The title compound C_12_H_14_S_3_O_2_ is a carbanotrithioate. It can be used as a chain transfer agent (CTA) in RAFT polymerization (Chong *et al.*, 1999) to control the polymerization and it will produce carbanotrithionate end-terminated polymers. Very few single-crystal XRD data are available for CTAs, because most of them are liquids (Coady *et al.*, 2008). Recently, we have reported the single-crystal data of a multi-functional CTA, which can be used for the synthesis of star polymers. Carbanotrithioate CTA is suitable for the polymerization of styrene, acrylates and methacrylates. With appropriate choice of the CTA (RAFT agent) and reaction conditions, RAFT polymerization can be successfully used to produce polymers of narrow polydispersity with predetermined molecular weights. Moreover, the polymers obtained by the RAFT process can be chain extended or used as precursors to synthesize stimuli responsive block copolymers by the addition of further monomer(*s*). The title compound will result in carboxylic acid end-terminated polymer; this functionality can be further modified and utilized for making block copolymers by reacting it with another homo-polymer.

The compound C_12_H_14_S_3_O_2_ is stabilized by a O—H···O interaction with *R*_2_^2^(8) graph set motif.

### Experimental

The title compound, was prepared by adding 3-mercapto propanoic acid (1.00 g, 7.35 mmol) to a stirred suspension of K_3_PO_4_ (1.72 g, 8.09 mmol) in acetone (20 ml) over a period of ten minutes. CS_2_ (1.68 g, 22.06 mmol) was added upon which the solution turned bright yellow. After stirring for ten minutes 1-bromo ethyl benzene (1.26 g, 7.35 mmol) was added and an instant precipitation of KBr was noted. After stirring for three hours the suspension was filtered and the cake was rinsed with acetone (2 × 20 ml). After removing the solvent from the filtrate under reduced pressure the resulting yellow residue was purified by column chromatography on silica using a petroleum ether/ethyl acetate gradient to yield light yellow solid (96%) that crystallized to form light yellow blocks.

### Refinement

All hydrogen atoms were fixed geometrically and allowed to ride on the parent carbon atoms, with aromatic C—H = 0.93 Å, methyl C—H = 0.96 Å and methylene C—H = 0.97 Å. The displacement parameters were set for phenyl and methylene H atoms at *U*_iso_(H) = 1.2*U*_eq_(C) and methyl H atoms at *U*_iso_(H) = 1.5*U*_eq_(C).

### Crystal data

**Table:**

C_12_H_14_O_2_S_3_	*F*(000) = 600
*M**_r_* = 286.41	*D*_x_ = 1.374 Mg m^−^^3^
Monoclinic, *P*2_1_/*c*	Mo *K*α radiation, λ = 0.71073 Å
Hall symbol: -P 2ybc	Cell parameters from 4284 reflections
*a* = 13.6280 (8) Å	θ = 2.6–27.2°
*b* = 10.2908 (5) Å	µ = 0.52 mm^−^^1^
*c* = 10.7299 (5) Å	*T* = 298 K
β = 113.039 (2)°	Block, light yellow
*V* = 1384.77 (12) Å^3^	0.42 × 0.28 × 0.22 mm
*Z* = 4	

### Data collection

**Table:**

Bruker APEXII CCD area-detector diffractometer	3020 independent reflections
Radiation source: fine-focus sealed tube	2224 reflections with *I* > 2σ(*I*)
graphite	*R*_int_ = 0.017
phi and ω scans	θ_max_ = 28.5°, θ_min_ = 2.6°
Absorption correction: multi-scan (*SADABS*; Bruker, 2004)	*h* = −17→18
*T*_min_ = 0.811, *T*_max_ = 0.894	*k* = −12→12
9109 measured reflections	*l* = −14→12

### Refinement

**Table:**

Refinement on *F*^2^	Primary atom site location: structure-invariant direct methods
Least-squares matrix: full	Secondary atom site location: difference Fourier map
*R*[*F*^2^ > 2σ(*F*^2^)] = 0.036	Hydrogen site location: inferred from neighbouring sites
*wR*(*F*^2^) = 0.095	H atoms treated by a mixture of independent and constrained refinement
*S* = 1.03	*w* = 1/[σ^2^(*F*_o_^2^) + (0.0372*P*)^2^ + 0.5628*P*] where *P* = (*F*_o_^2^ + 2*F*_c_^2^)/3
3020 reflections	(Δ/σ)_max_ = 0.001
159 parameters	Δρ_max_ = 0.32 e Å^−^^3^
0 restraints	Δρ_min_ = −0.27 e Å^−^^3^

### Special details

**Table:**

Geometry. All e.s.d.'s (except the e.s.d. in the dihedral angle between two l.s. planes)are estimated using the full covariance matrix. The cell e.s.d.'s are takeninto account individually in the estimation of e.s.d.'s in distances, anglesand torsion angles; correlations between e.s.d.'s in cell parameters are onlyused when they are defined by crystal symmetry. An approximate (isotropic)treatment of cell e.s.d.'s is used for estimating e.s.d.'s involving l.s. planes.
Refinement. Refinement of *F*^2^ against ALL reflections. The weighted *R*-factor *wR* and goodness of fit *S* are based on *F*^2^, conventional *R*-factors *R* are based on *F*, with *F* set to zero for negative *F*^2^. The threshold expression of *F*^2^ > σ(*F*^2^) is used only for calculating *R*-factors(gt) *etc*. and is not relevant to the choice of reflections for refinement. *R*-factors based on *F*^2^ are statistically about twice as large as those based on *F*, and *R*- factors based on ALL data will be even larger.

### Fractional atomic coordinates and isotropic or equivalent isotropic displacement parameters (Å^2^)

**Table:**

	*x*	*y*	*z*	*U*_iso_*/*U*_eq_	
C1	0.34830 (19)	0.5248 (2)	0.1937 (3)	0.0664 (6)	
H1	0.3225	0.5785	0.1180	0.080*	
C2	0.4227 (2)	0.4306 (3)	0.2022 (4)	0.0874 (9)	
H2	0.4469	0.4216	0.1328	0.105*	
C3	0.4606 (2)	0.3509 (3)	0.3116 (4)	0.0948 (11)	
H3	0.5103	0.2870	0.3168	0.114*	
C4	0.4260 (2)	0.3645 (3)	0.4129 (4)	0.0912 (10)	
H4	0.4519	0.3095	0.4875	0.109*	
C5	0.3523 (2)	0.4598 (2)	0.4070 (3)	0.0684 (6)	
H5	0.3302	0.4692	0.4783	0.082*	
C6	0.31156 (16)	0.54066 (18)	0.2959 (2)	0.0492 (5)	
C7	0.23156 (16)	0.64611 (17)	0.2845 (2)	0.0477 (5)	
H7	0.1806	0.6504	0.1901	0.057*	
C8	0.1697 (2)	0.6301 (3)	0.3737 (3)	0.0832 (8)	
H8A	0.1330	0.5482	0.3546	0.125*	
H8B	0.1187	0.6993	0.3559	0.125*	
H8C	0.2180	0.6327	0.4671	0.125*	
C9	0.21603 (15)	0.91964 (17)	0.24871 (19)	0.0421 (4)	
C10	0.18484 (18)	1.18835 (19)	0.2244 (2)	0.0553 (5)	
H10A	0.1186	1.1527	0.2231	0.066*	
H10B	0.2046	1.2608	0.2872	0.066*	
C11	0.16642 (16)	1.23796 (18)	0.0855 (2)	0.0479 (5)	
H11A	0.2342	1.2624	0.0825	0.057*	
H11B	0.1359	1.1692	0.0199	0.057*	
C12	0.09317 (15)	1.35291 (18)	0.0482 (2)	0.0444 (4)	
O1	0.04244 (14)	1.38506 (15)	0.11389 (17)	0.0706 (5)	
O2	0.08929 (15)	1.41368 (17)	−0.05809 (18)	0.0708 (5)	
S1	0.30835 (4)	0.79721 (5)	0.32472 (6)	0.05737 (18)	
S2	0.09018 (4)	0.90297 (6)	0.15929 (6)	0.06006 (18)	
S3	0.28670 (5)	1.06576 (5)	0.28523 (7)	0.06266 (19)	
H1O	0.051 (3)	1.477 (3)	−0.073 (3)	0.111 (12)*	

### Atomic displacement parameters (Å^2^)

**Table:**

	*U*^11^	*U*^22^	*U*^33^	*U*^12^	*U*^13^	*U*^23^
C1	0.0664 (15)	0.0545 (13)	0.0817 (17)	0.0057 (11)	0.0326 (13)	−0.0060 (12)
C2	0.0686 (18)	0.0723 (18)	0.125 (3)	0.0037 (14)	0.0424 (18)	−0.0282 (18)
C3	0.0550 (17)	0.0490 (15)	0.163 (3)	0.0051 (12)	0.023 (2)	−0.0112 (18)
C4	0.0649 (18)	0.0499 (15)	0.127 (3)	−0.0007 (13)	0.0035 (18)	0.0247 (16)
C5	0.0650 (15)	0.0477 (13)	0.0813 (17)	−0.0060 (11)	0.0166 (13)	0.0118 (12)
C6	0.0460 (12)	0.0316 (10)	0.0666 (14)	−0.0063 (8)	0.0182 (10)	−0.0023 (9)
C7	0.0498 (12)	0.0357 (10)	0.0610 (13)	−0.0039 (8)	0.0256 (10)	0.0005 (9)
C8	0.098 (2)	0.0674 (16)	0.115 (2)	0.0002 (15)	0.0750 (19)	0.0082 (15)
C9	0.0449 (11)	0.0387 (10)	0.0425 (11)	0.0042 (8)	0.0170 (9)	0.0011 (8)
C10	0.0641 (14)	0.0361 (10)	0.0645 (14)	0.0124 (9)	0.0240 (11)	0.0065 (9)
C11	0.0471 (11)	0.0399 (10)	0.0585 (13)	0.0088 (8)	0.0228 (10)	0.0041 (9)
C12	0.0457 (11)	0.0380 (10)	0.0515 (12)	0.0057 (8)	0.0214 (10)	0.0048 (8)
O1	0.0899 (12)	0.0649 (10)	0.0778 (11)	0.0397 (9)	0.0553 (10)	0.0286 (8)
O2	0.0879 (13)	0.0675 (11)	0.0748 (12)	0.0384 (10)	0.0510 (10)	0.0314 (9)
S1	0.0441 (3)	0.0327 (3)	0.0829 (4)	0.0021 (2)	0.0114 (3)	0.0055 (2)
S2	0.0411 (3)	0.0620 (3)	0.0680 (4)	0.0046 (2)	0.0115 (3)	−0.0083 (3)
S3	0.0507 (3)	0.0354 (3)	0.0857 (5)	0.0032 (2)	0.0092 (3)	0.0112 (3)

### Geometric parameters (Å, °)

**Table:**

C1—C2	1.380 (3)	C8—H8B	0.9600
C1—C6	1.383 (3)	C8—H8C	0.9600
C1—H1	0.9300	C9—S2	1.614 (2)
C2—C3	1.357 (5)	C9—S1	1.7415 (19)
C2—H2	0.9300	C9—S3	1.7455 (19)
C3—C4	1.351 (5)	C10—C11	1.500 (3)
C3—H3	0.9300	C10—S3	1.799 (2)
C4—C5	1.388 (4)	C10—H10A	0.9700
C4—H4	0.9300	C10—H10B	0.9700
C5—C6	1.380 (3)	C11—C12	1.498 (2)
C5—H5	0.9300	C11—H11A	0.9700
C6—C7	1.509 (3)	C11—H11B	0.9700
C7—C8	1.512 (3)	C12—O1	1.211 (2)
C7—S1	1.8291 (19)	C12—O2	1.283 (2)
C7—H7	0.9800	O2—H1O	0.81 (3)
C8—H8A	0.9600		
			
C2—C1—C6	121.0 (3)	H8A—C8—H8B	109.5
C2—C1—H1	119.5	C7—C8—H8C	109.5
C6—C1—H1	119.5	H8A—C8—H8C	109.5
C3—C2—C1	120.2 (3)	H8B—C8—H8C	109.5
C3—C2—H2	119.9	S2—C9—S1	127.34 (11)
C1—C2—H2	119.9	S2—C9—S3	126.14 (11)
C4—C3—C2	119.9 (3)	S1—C9—S3	106.51 (11)
C4—C3—H3	120.0	C11—C10—S3	113.87 (14)
C2—C3—H3	120.0	C11—C10—H10A	108.8
C3—C4—C5	120.8 (3)	S3—C10—H10A	108.8
C3—C4—H4	119.6	C11—C10—H10B	108.8
C5—C4—H4	119.6	S3—C10—H10B	108.8
C6—C5—C4	120.3 (3)	H10A—C10—H10B	107.7
C6—C5—H5	119.9	C12—C11—C10	111.69 (16)
C4—C5—H5	119.9	C12—C11—H11A	109.3
C5—C6—C1	117.8 (2)	C10—C11—H11A	109.3
C5—C6—C7	122.5 (2)	C12—C11—H11B	109.3
C1—C6—C7	119.66 (19)	C10—C11—H11B	109.3
C6—C7—C8	115.77 (18)	H11A—C11—H11B	107.9
C6—C7—S1	105.29 (13)	O1—C12—O2	123.36 (18)
C8—C7—S1	110.65 (16)	O1—C12—C11	122.18 (17)
C6—C7—H7	108.3	O2—C12—C11	114.46 (16)
C8—C7—H7	108.3	C12—O2—H1O	112 (2)
S1—C7—H7	108.3	C9—S1—C7	105.23 (9)
C7—C8—H8A	109.5	C9—S3—C10	104.07 (10)
C7—C8—H8B	109.5		
			
C6—C1—C2—C3	0.4 (4)	C1—C6—C7—S1	−76.6 (2)
C1—C2—C3—C4	−0.5 (4)	S3—C10—C11—C12	−171.50 (14)
C2—C3—C4—C5	−0.3 (4)	C10—C11—C12—O1	−11.6 (3)
C3—C4—C5—C6	1.2 (4)	C10—C11—C12—O2	168.19 (19)
C4—C5—C6—C1	−1.3 (3)	S2—C9—S1—C7	−1.18 (16)
C4—C5—C6—C7	−179.8 (2)	S3—C9—S1—C7	179.94 (9)
C2—C1—C6—C5	0.5 (3)	C6—C7—S1—C9	156.85 (14)
C2—C1—C6—C7	179.0 (2)	C8—C7—S1—C9	−77.38 (19)
C5—C6—C7—C8	−20.6 (3)	S2—C9—S3—C10	8.69 (16)
C1—C6—C7—C8	160.9 (2)	S1—C9—S3—C10	−172.41 (10)
C5—C6—C7—S1	101.9 (2)	C11—C10—S3—C9	−97.04 (17)

### Hydrogen-bond geometry (Å, °)

**Table:**

*D*—H···*A*	*D*—H	H···*A*	*D*···*A*	*D*—H···*A*
O2—H1O···O1^i^	0.81 (3)	1.85 (3)	2.651 (2)	177 (3)

Symmetry codes: (i) −*x*, −*y*+3, −*z*.
